# *Pseudomonas aeruginosa* bloodstream infection at a tertiary referral hospital for children

**DOI:** 10.1186/s12879-020-05437-1

**Published:** 2020-10-07

**Authors:** Joycelyn Assimeng Dame, Natalie Beylis, James Nuttall, Brian Eley

**Affiliations:** 1 Paediatric Infectious Diseases Unit, Red Cross War Memorial Children’s Hospital and the Department of Paediatrics and Child Health, University of Cape Town, Cape Town, South Africa; 2 National Health Laboratory Service, Groote Schuur Hospital and Division of Medical Microbiology, University of Cape Town, Cape Town, South Africa

**Keywords:** *Pseudomonas aeruginosa* bloodstream infection, Children, Sub-Saharan Africa

## Abstract

**Background:**

This study describes the disease burden, clinical characteristics, antibiotic management, impact of multidrug resistance and outcome of *Pseudomonas aeruginosa* bloodstream infection (PABSI) among children admitted to a tertiary referral hospital for children in Cape Town, South Africa.

**Methods:**

A retrospective descriptive study was conducted at a paediatric referral hospital in Cape Town, South Africa. Demographic and clinical details, antibiotic management and patient outcome information were extracted from medical and laboratory records. Antibiotic susceptibility results of identified organisms were obtained from the National Health Laboratory Service database.

**Results:**

The incidence risk of PABSI was 5.4 (95% CI: 4.34–6.54) PABSI episodes / 10,000 hospital admissions and the most common presenting feature was respiratory distress, 34/91 (37.4%). Overall, 69/91 (75.8%) of the PA isolates were susceptible to all antipseudomonal antibiotic classes evaluated. Fifty (54.9%) of the PABSI episodes were treated with appropriate empiric antibiotic therapy. The mortality rate was 24.2% and in multivariable analysis, empiric antibiotic therapy to which PA isolates were not susceptible, infections present on admission, and not being in the intensive care unit at the time that PABSI was diagnosed were significantly associated with 14-day mortality.

**Conclusions:**

PABSI caused appreciable mortality, however, appropriate empiric antibiotic therapy was associated with reduced 14-day mortality.

## Background

*Pseudomonas aeruginosa* (PA) is a ubiquitous Gram-negative bacterium usually found in water, soil and plants. Studies from South Africa and Ghana have shown that it causes between 4 and 6.5% of Gram-negative BSI [1–3]. PA typically causes healthcare-associated BSI among children with chronic or malignant diseases that are associated with impaired defence mechanisms [4, 5]. Community-acquired PA bloodstream infection (PABSI) may manifest in children with other immunodeficiency states including hypogammaglobulinaemia and neutropaenia [6–8]. Community-acquired PABSI has also been reported among previously healthy, young children without underlying medical conditions [9, 10].

The mortality of PABSI is high. In retrospective studies from Argentina and Taiwan, case-fatality rates of 30 and 35% respectively were documented [9, 11]. Risk factors for mortality in children with PABSI include septic shock, multidrug-resistant (MDR) PA isolates, admission to an intensive care unit (ICU), the presence of an underlying disease, a pulmonary source of infection, inappropriate empiric antibiotic therapy and diarrhoea as a presenting feature [11–14].

PA is intrinsically resistant to certain commonly used beta-lactam antibiotics such as ampicillin and ceftriaxone and can acquire resistance during therapy to other antibiotics such as the carbapenems [15, 16]. This makes the selection of empiric antibiotic therapy for suspected PABSI challenging. In a study done at a Korean university hospital in 75 children with PABSI, the prevalence of MDR PA was 11.3%. In that study, the fatality rate was higher among children with PABSI caused by MDR isolates compared to those with non-MDR PA isolates, 57.1% versus 9.1% [17].

While previous studies from sub-Saharan Africa have reported on the prevalence of PA in children with BSI, there are no paediatric studies providing detailed description of PABSI in children in sub-Saharan Africa. The present study was undertaken to address this knowledge gap.

## Methods

### Aim

The aim of this study was to describe the disease burden, clinical characteristics, antibiotic management, impact of multidrug resistance and outcome of PABSI among children admitted to a tertiary referral hospital for children in Cape Town, South Africa.

### Study design, setting and inclusion criteria

This retrospective descriptive study was conducted at Red Cross War Memorial Children’s Hospital (RCWMCH), Cape Town, South Africa. This 273-bed facility serves as a referral centre for the paediatric population of the Western Cape province as well as surrounding provinces. Hospitalised children aged 0 to 14 years with culture- confirmed PABSI that was diagnosed between January 2009 and December 2017 were included in the analysis. Repeat blood culture results and PABSI episodes with insufficient antibiotic and/or clinical information were excluded from the analysis.

### Data collection

The Central Data Warehouse (CDW), housed in the information technology department of the National Health Laboratory Service (NHLS) in Johannesburg, South Africa, is a database of all laboratory investigations performed on patients treated in public sector hospitals and clinics in South Africa. The academic and research unit managing the CDW retrieved the list of children admitted at RCWMCH with laboratory- confirmed PABSI from January 2009 until December 2017. This list was used to obtain microbiology results relating to every PABSI episode during the study period. These microbiology results were extracted from the NHLS microbiology database at Groote Schuur Hospital (GSH), Cape Town. Blood culture specimens from children admitted to RCWMCH are routinely transported to the GSH microbiology laboratory where they are processed. Clinical data relating to each PABSI episode were extracted from the patient hospital records at RCWMCH. All microbiology and clinical data were entered in study-specific data collection sheets.

### Microbiology testing

All microbiology testing was conducted at the NHLS clinical microbiology laboratory based at GSH. From 2009 to 2013, the BACTEC 9240 automated blood culture system (Becton Dickinson, Sparks, MD, USA) with BACTEC Plus aerobic blood culture bottles was in use. From 2013 until the end of the study period, BacT/ALERT PF Plus aerobic paediatric bottles were used along with the BacT/ALERT automated blood culture system (bioMerieux Inc., Durham, NC, USA).

Bacterial identification and antibiotic susceptibility testing were performed according to the results of the Gram stain with final identification by the automated Vitek®2 system (bioMérieux, Inc., France) for the duration of the study period. Vitek®2ID-GNB card was used for identification testing throughout the study period; the susceptibility testing card used by the laboratory was N133 until 2012; N064 until 2014 and N255 until 2017. All of the Vitek® 2 AST cards used during the study period include the following anti-pseudomonal antibiotics: amikacin, gentamicin, ceftazidime, cefepime, ciprofloxacin, piperacillin-tazobactam, tigecycline, meropenem, imipenem and colistin.

The antibiotic susceptibility testing methods and breakpoints were based on the Clinical and Laboratory Standards Institute (CLSI) of each year of the study [18]. Changes in 2012 included an increase in the susceptibility breakpoints for piperacillin-tazobactam from ≤64/4 μg/mL to ≤16/4 μg/mL, and the introduction of a new intermediate category for piperacillin-tazobactam from 32/4 μg/mL to 64/4 μg/mL. In addition, the susceptible category breakpoints for imipenem and meropenem changed from ≤4 μg/mL to ≤2 μg/mL in 2012, while the intermediate category range changed from 8 to 4 μg/mL. Breakpoints for all anti-pseudomonal antibiotics remained the same from 2012 until the end of the study period. The CLSI changes in breakpoints were incorporated as updates in the Vitek®2 interpretation software when they occurred. The *Pseudomonas aeruginosa* ATCC 27853 control strain was used during routine Vitek® 2 testing throughout the study period.

### Statistical analysis

The data was analysed using STATA Statistical software, release 11, (College Station, Texas, USA). Incidence risk of PA-BSI was calculated per 10,000 hospital admissions. Proportions were depicted as percentages. Continuous variables were tested for normality and mean and standard deviation (SD) or median and interquartile range (IQR) used to describe the data as appropriate. The Student’s t- test or Mann Whitney U test were used to compare continuous data based on their normal distribution, whilst the chi-squared, and Fisher’s exact test was used to compare categorical data. A two-sided significance level of *p* < 0.05 was considered statistically significant. Predictors of 14-day mortality were explored using univariable and multivariable logistic regression analyses. The logistic regression model was built by stepwise backward selection, incorporating variables which on univariable analysis had a *p* value < 0.20. The results of the logistic regression model were expressed as adjusted odds ratio (aOR) and 95% confidence intervals (CIs).

Definitions – refer Supplementary file.

## Results

### Study participants

**Fig. 1:**
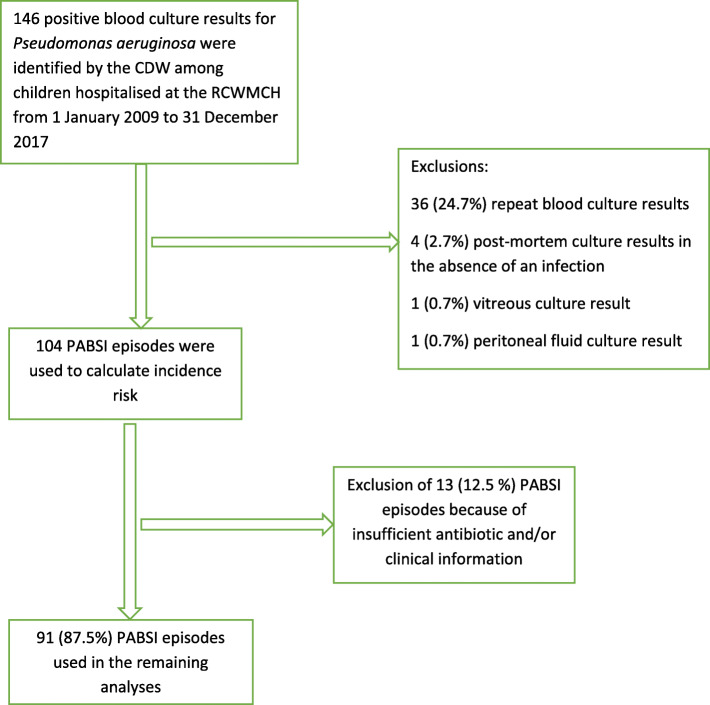
Selection of *Pseudomonas aeruginosa* bloodstream infection episodes for data analysis. CDW, Central Data warehouse; PABSI, *Pseudomonas aeruginosa* bloodstream infection; RCWMCH, Red Cross War Memorial Children’s Hospital

During the study period there were 192,547 admissions to RCWMCH and 104 PABSI episodes. These episodes were used to estimate the risk of PABSI. Thirteen episodes of PABSI were excluded due to insufficient antibiotic and/or clinical data, thus 91 (88.0%) children were used in the subsequent analyses (Fig. 1).

### Classification and risk of *Pseudomonas aeruginosa* BSI

**Fig. 2:**
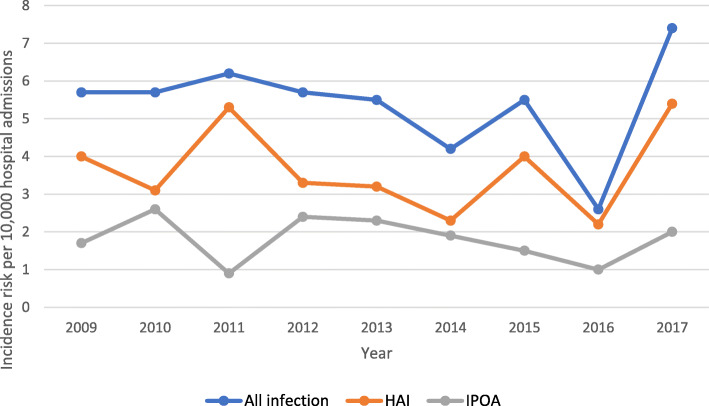
Annual incidence risk per 10,000 hospital admissions. HAI, healthcare-associated infection; IPOA, infection present on admission

Out of 104 PABSI episodes, 69 (66.3%) were HAIs and 35 (33.7%) were IPOA. There was one recurrent PABSI episode, a HAI which manifested 30 days after the initial PABSI episode. The overall incidence risk of PABSI throughout the study period was 5.4 (95% CI: 4.34–6.54) PABSI episodes / 10,000 hospital admissions. There was a decline in annual incidence risk from 2009 until 2016 followed by a rise in 2017. This increase was mainly related to an increase in the incidence risk of HAI (Fig. 2). The annual incidence risk of HAI was consistently higher than IPOA throughout the study period.

### Characteristics of study participants

Table 1 describes characteristics of 91 PABSI episodes in 91 study participants, 60 (65.9%) episodes were HAIs and 31 (34.1%) were IPOA. The median time between admission and the development of PABSI in the 60 children who developed HAI was 13.5 days (IQR 7.0–28.0). The median age was 12 months (IQR 5–59) and 52% of the episodes occurred in females. Eleven of the 15 children with chronic diseases other than HIV infection had malignancies, namely, acute lymphoblastic leukaemia (3), acute myeloid leukaemia (2), lymphoma (1), neuroblastoma (1), craniopharyngioma (1) and germ cell tumour (1). Other chronic diseases included chronic lung disease (2), Fanconi anaemia (1) and aplastic anaemia (1).

**Table 1:** Characteristics of children at the time of *Pseudomonas aeruginosa* bloodstream infection

Variable	Total N = 91	HAI N = 60	IPOA N = 31	*P* value*
Age (months) median (IQR)	12 (5–59)	10 (4.5–46)	19 (6–69)	0.065
Age category, n/N (%)				
*< 1 year*	45 (49.5)	34 (56.7)	11 (35.5)	0.107
*1–5 years*	22 (24.2)	11 (18.3)	11 (35.5)	
*> 5 years*	24 (26.3)	15 (25.0)	9 (29.0)	
Gender, n/N (%)				
*Male*	44 (48.4)	27 (45.0)	17 (55.8)	0.387
*Female*	47 (51.6)	33 (55.0)	14 (45.2)	
HIV status, n/N (%)				
*HIV-infected*	13 (14.3)	3 (5.0)	10 (32.3)	0.002
*HIV-uninfected*	51 (56.0)	36 (60.0)	15 (48.4)	
*Unknown*	27 (29.7)	21 (35.0)	6 (19.4)	
Weight-for-age, Z score category, n/N (%)				
*Moderate underweight*	9 (10.2)	3 (5.2)	6 (20.0)	0.008
*Severe underweight*	22 (25.0)	11 (19.0)	11 (36.7)	
Temperature in degrees Celsius				
*< 35 °C*	3 (3.4)	1 (1.8)	2 (6.7)	0.459
*> 35.5–37.9 °C*	19 (21.8)	12 (21.1)	7 (23.3)	
*≥ 38.0 °C*	65 (74.7)	44 (77.2)	21 (70.0))	
Anaemia	64 (70.3)	40 (66.7)	24 (77.4)	0.210
Hospitalisation in the 28-day period preceding the current admission, n/N (%)	47 (51.6)	27 (45.0)	20 (64.5)	0.121
Exposure to selective intravenous antibiotics, in the preceding 12 months, n/N (%)	47 (51.7)	38 (63.3)	9 (29.0)	0.004
Selective intravenous antibiotics exposure in the preceding 12 months, n/N (%)				
*Gentamicin or amikacin*	23 (25.3)	19 (31.7)	4 (12.4)	0.074
*2nd to 4th generation cephalosporins*	13 (14.3)	8 (61.5)	5 (38.5)	0.757
*Piperacillin-tazobactam*	12 (13.2)	12 (20.0)	0 (0)	0.005
*Meropenem or ertapenem*	16 (17.6)	15 (25.0)	1 (3.2)	0.009
Chronic diseases other than HIV infection, n/N (%)	15 (16.5)	6 (10.0)	9 (29.0)	0.034
ICU admission prior to PABSI, n/N (%)	61 (67.0)	50 (83.3)	11 (35.3)	0.0001
Central venous access device in situ, n/N (%)	59 (54.8)	48 (80.0)	11 (35.5)	0.0001
Endotracheal Intubation in situ, n/N (%)	54 (59.3)	46 (76.7)	8 (25.8)	0.0001
Burn wound, n/N (%)	19 (21.1)	11 (18.3)	8 (26.7)	0.416
Surgery during current admission, n/N (%)	46 (51.1)	39 (65.0)	7 (23.3)	0.0001

*Comparison of HAI and IPOA, *HAI* Healthcare-associated infection, *IPOA* Infections present on admission, *PABSI Pseudomonas aeruginosa* bloodstream infection, *C* Celsius, *ICU* Intensive care unit

### Presenting clinical features and complications of PABSI

Respiratory distress was the commonest presenting feature overall (34/91, 37.4%) and for HAIs (23/60, 38.3%) whereas diarrhoea was the commonest presenting feature for IPOA (13/31, 41.9%) (Table 2).

**Table 2:** Presenting clinical features of *Pseudomonas aeruginosa* bloodstream infection and site of infection

Variable	Total *N* = 91 n/N (%)	HAI *N* = 60 n/N (%)	IPOA *N* = 31 n/N (%)	*P* value
Presenting features^a^				
*Respiratory distress*	34 (37.4)	23 (38.3)	11 (35.5)	0.487
*Diarrhoea*	29 (31.9)	16 (26.7)	13 (41.9)	0.107
*Wound infection*	23 (25.3)	18 (30.0)	5 (16.1)	0.116
*Shock*	13 (14.3)	4 (6.7)	9 (29.0)	0.009
*Ecthyma gangrenosum*	3 (3.3)	1 (1.7)	2 (6.5)	0.267
*Otitis media*	4 (4.4)	1 (1.7)	3 (9.7)	0.113
*Other*^b^	7 (7.7)	3 (5.0)	4 (12.9)	0.224
Site of infection				
*No definable focus*	18 (19.8)	12 (20.0)	6 (19.4)	1.000
*Pneumonia*	33 (36.3)	22 (36.7)	11 (35.5)	1.000
*Gastrointestinal tract*^c^	7 (7.7)	3 (5.0)	4 (12.9)	0.224
*Skin & soft tissue infection*	20 (22.0)	16 (26.7)	4 (12.9)	0.184
*Line infection*	8 (8.8)	6 (10.0)	2 (6.5)	0.711
*Urosepsis*	4 (4.4)	1 (1.7)	3 (9.7)	0.132

*HAI* Healthcare-associated infection, *IPOA* Infection present on admission; a, some patients presented with > 1 presenting feature; b, vomiting (2), renal angle tenderness (1), necrotising bowel (1), tachycardia (1), eye discharge (1), acute abdomen (1); c, gastroenteritis (4), acute appendicitis (1), acute peritonitis (2).

### Complications of PABSI

Shock, as determined by the attending clinicians, was the most common complication, occurring in 16/91 (17.6%) of the children; 11/16 (68.8%) required inotropic infusions. Shock, liver dysfunction and 14-day mortality were significantly more frequent complications in children with IPOA (Table 3).

**Table 3:** Complications and outcome associated with *Pseudomonas aeruginosa* bloodstream infection

Variable	Total N = 91 n/N (%)	HAI N = 60 n/N (%)	IPOA N = 31 n/N (%)	*P* value
Shock	16 (17.6)	7 (11.7)	9 (29.0)	0.047
Coagulopathy	10 (11.1)	5 (8.3)	5 (16.1)	0.300
Renal dysfunction	12 (13.2)	7 (11.7)	5 (16.1)	0.534
Liver dysfunction	4 (4.4)	0 (0)	4 (12.9)	0.012
Respiratory failure	11 (12.1)	6 (10.0)	5 (16.1)	0.500
14-day mortality	17 (18.7)	6 (10.0)	11 (35.5)	0.005

*HAI* Healthcare-associated infection, *IPOA* Infection present on admission.

### Susceptibility profile of PA isolates and antibiotic therapy

**Fig. 3:**
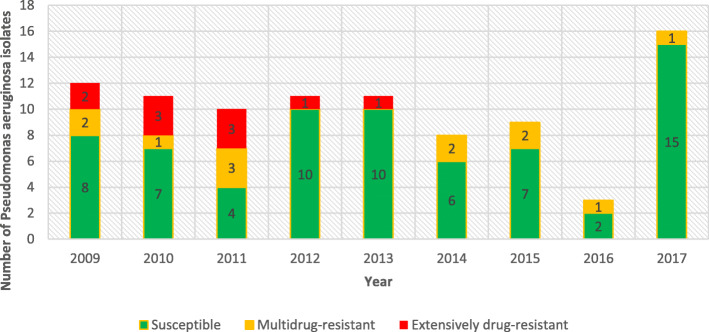
Annual antibiotic susceptibility profile of *Pseudomonas aeruginosa* isolates by antipseudomonal antibiotic susceptibility category, 2009–2017. M*ultidrug-resistant (MDR)*, non-susceptible to at least one agent in three or more antipseudomonal antibiotic categories; *extensively drug-resistant (XDR)*, non-susceptible to at least one agent in all but two or fewer antipseudomonal antibiotic categories

Figure 3 summarises the susceptibility profile of the PA isolates during the study period. Overall, 69/91 (75.8%) of the PA isolates were susceptible to all antipseudomonal antibiotic categories evaluated. There were 12/91 (13.2%) MDR isolates and 10/91 (11.0%) XDR isolates. The proportion of HAI isolates amongst the resistant isolates was as follows; 8/12 (66.7%) MDR and 9/10 (90.0%) XDR isolates.

There were 19/91 (20.9%) isolates that were resistant to both imipenem and meropenem; there were an additional 2 that were resistant only to imipenem but not to meropenem i.e. a total of 21/91 (23.1%) isolates were resistant to imipenem. Ten meropenem-resistant isolates were susceptible to ceftazidime, (10/91; 9.1%), while 4/91 (4.4%) ceftazidime-resistant isolates were susceptible to meropenem. There were 8/91 (8.8%) isolates that were resistant to both ceftazidime and meropenem.

Fifty (54.9%) of the PABSI episodes were treated with appropriate empiric antibiotic therapy. A higher proportion of HAI PABSI episodes received appropriate empiric antibiotic therapy compared to IPOA PABSI episodes; 37/60 (61.7%) versus 13/31 (41.9%). This difference was, non-significant, *p* = 0.081. Three antibiotics frequently used in empiric therapy for both HAI and IPOA were meropenem 27/91(29.7%), piperacillin-tazobactam 19/91(20.9%), amikacin 18/91 (19.8%). Piperacillin-tazobactam was frequently combined with amikacin for empiric therapy 18/19 (94.7%).

The mean time ± SD to effective antibiotic therapy (appropriate empiric antibiotic therapy or definitive antibiotic therapy) was 1.3 days ±1.1. The difference in mean time to effective antibiotic therapy for HAI was 1.2 days ±0.9 and 1.6 days ±1.5 in IPOA, this was not significant (*p* = 0.112). Overall, 37/91 (40.7%) PABSI episodes were treated with meropenem; 23 of these 37 episodes (62.2%) isolates were caused by isolates that were susceptible to ceftazidime. By contrast, only 17.6% of the PABSI episodes were treated with ceftazidime (Table 4).

**Table 4:** Antibiotic susceptibility of *Pseudomonas aeruginosa* bloodstream infection isolates, and the definitive antibiotic therapy used during the study period

Antibiotic	Susceptibility of PA isolates to anti-pseudomonal antibiotics			Antibiotics used as definitive antibiotic therapy		
	HAI *N* = 60 n/N (%)	IPOA *N* = 31 n/N (%)	Total *N* = 91 n/N (%)	HAI N = 60 n/N (%)	IPOA N = 31 n/N (%)	Total *N* = 91 n/N (%)
Gentamicin	43 (71.7)	28 (90.3)	71 (78.0)	5 (8.3)	2 (6.5)	7 (7.7)
Amikacin	50 (83.3)	28 (90.3)	78 (85.7)	10 (16.7)	6 (19.4)	16 (17.6)
Ciprofloxacin	42 (70.0)	28 (90.3)	70 (76.9)	12 (20.0)	7 (22.6)	19 (20.9)
Piperacillin-tazobactam	35 (58.3)	19 (61.3)	54 (59.3)	12 (20.0)	9 (29.0)	21 (23.1)
Ceftazidime	50 (83.3)	29 (93.5)	79 (86.8)	8 (13.3)	8 (25.8)	16 (17.6)
Cefepime	47 (78.3	26 (83.9)	73 (80.2)	11 (18.3)	2 (6.5)	13 (14.3)
Meropenem	44 (73.3)	28 (90.3)	72 (79.1)	27 (45.0)	10 (32.3)	37 (40.7)
Imipenem Colistin	43 (71.7) -	27 (87.1) -	70 (76.9) -	2 (3.3) 9 (15)	0 (0) -	2 (2.2) 9 (9.9)

*PA Pseudomonas aeruginosa*, *HAI* Healthcare-associated infection, *IPOA* Infection present on admission.

### Outcome

There were 69/91 (75.8%) PABSI episodes that were successfully treated. Twenty-two (24.2%) of the children died during hospitalisation. Most of the deaths, 17/22 (77.3%), occurred within 14 days of hospitalisation as a direct result of PABSI. The median time (IQR) to death was 1.4 (1.0–8.3) days, and 11/17 (64.7%) of these deaths were due to IPOA. Of the 5 deaths that occurred after 14 days, the median time (IQR) to death was 22.5 (20.8–30.3) days and 3/5 (60.0%) had IPOA.

On multivariable analysis, empiric antibiotic therapy to which PA isolate was not susceptible to, IPOA, and not being admitted in the ICU at the time that PABSI was diagnosed were significantly associated with 14-day mortality. (see Table 5).

**Table 5:** Predictors of 14-day mortality in children with *Pseudomonas aeruginosa* bloodstream infection

Variable	Unadjusted OR (95% confidence interval)	*P*-value	Adjusted OR (95% confidence interval)	*P* value
Age category N = 91				
*< 1 year*	1		_	
*≥ 1 year*	0.66 (0.23–1.93)	0.45		
Weight *N* = 88				
*Normal weight*	1			
*Moderate or severe underweight*	0.44 (0.15–1.32)	0.10	1.60 (0.330–7.803)	0.557
Admission in a health care facility 28 days prior to current hospitalisation	0.80 (0.27–2.4)	0.79	_	
Chronic disease (excluding HIV) *N* = 80	1.2 (0.24–6.13)	0.827	_	
HIV status				
*HIV-uninfected*	1			
*HIV-infected*	1.34 (0.32–5.68)	0.70	_	
*HIV status unknown*	0.89 (0.22–3.62)	0.87		
Nature of infection				
*IPOA*	1			
*HAI*	0.202 (0.066–0.619)	0.005	0.083 (0.01–0.60)	0.013
MDR, XDR or PDR isolates	1.05 (0.30–3.61)	0.945	_	
Appropriate empiric antibiotic	0.19 (0.06–0.63)	0.007	0.23 (0.07–0.82)	0.023
Presence of a central venous assess device	1.5(0.46–5.06)	0.495	–	
Burn wounds	2.24 (0.47–10.77)	0.315	_	
Septic shock	3.29 (1.05–10.23)	0.040	2.27 (0.41–12.55)	0.346
Presence of any organ dysfunction	1.97(0.61–6.44)	0.261	–	
Anaemia	1.47 (0.43–4.99)	0.540	–	
Surgery during current admission	1.96 (0.64–6.0)	0.235	–	
Presence of a central venous access device	1.52(0.46–5.06)	0.495	–	
ICU management				
*Patient in the ICU prior to PABSI diagnosis*	0.43 (0.15–1.25)	0.101	0.04 (0.005–0.365)	0.004
*Patient requiring ICU care after PABSI diagnosis*	1.21 (0.399–3.670)	0.737	–	
Number of antipseudomonal antibiotics used as definitive therapy				
*1 antipseudomonal antibiotic*	1			
*2 or more antipseudomonal antibiotics*	1.236 (0.345–4.430)	0.745		

*OR* Odds ratio, *HAI* Healthcare-associated infection, *IPOA* Infection present on admission, *MDR* Multidrug-resistant, *XDR* Extensively drug-resistant, *PDR* Pan drug-resistant

## Discussion

This retrospective descriptive study was conducted on laboratory confirmed PABSI in children admitted to RCWMCH between 1 January 2009 and 31 December 2017. To the best of our knowledge, this is the first study from sub-Saharan Africa describing PABSI in detail among hospitalised children. The majority of the PABSI episodes, 69/104(66.3%) were HAIs. This is consistent with previous research that showed that PA infections are mostly healthcare-associated [11, 17]. Environmental analysis suggests that PA is found in the moist areas of hospitals such as sinks and colonises respiratory equipment in Intensive Care Units (ICUs) [13], highlighting the importance of good infection prevention and control practices in preventing healthcare-associated PABSI.

In addition to hospital admission, host factors are important determinants of infection. The prevalence of underlying chronic diseases and HIV infection were significantly higher in children with IPOA in this study. In these children, impaired host defence mechanisms are likely to increase the risk of PABSI as described in previous studies [12, 19]. The CDC definition for IPOA used in the present study includes all infections in whom the corresponding isolates were cultured on the day of admission, 2 days before admission or the day after admission, irrespective of a recent hospitalisation. In the present study 20/31 (64.5%) of the PABSI episodes classified as IPOA were preceded by hospitalisation occurring within 28 days prior to the current hospitalization, suggesting that prior exposure to the hospital environment may have led to PA colonisation of the children, and effective IPC practices may have prevented some of these PABSI episodes.

PABSI can present with common childhood symptoms of a febrile illness such as respiratory distress, diarrhoea and discharging ears or with severe manifestations such as septic shock [4, 19]. In this study, respiratory distress and diarrhoea were the most common presenting symptoms.

Healthcare-associated infections were significantly more frequent in children who developed PABSI in the ICU, those with a central venous access device and/or endotracheal tube, and those who had had surgery during the current admission. Patients treated in the ICU tend to be immunocompromised, require invasive procedures and may be exposed to broad-spectrum antibiotics, and in this study those who had received intravenous broad-spectrum antibiotics such as piperacillin-tazobactam or meropenem 12 months prior to the current admission had significantly more HAI than IPOA. Implementation of appropriate infection prevention and control intervention bundles and antimicrobial stewardship must be part of ICU management to decrease the incidence of HAI caused by PA.

The overall in-hospital mortality rate of 24.2% was lower than reported in previous paediatric studies. At a single centre in China, the overall mortality rate was 52.0% over a 5-year period among 36 children, with a significant association between mortality and ineffective initial antibiotic therapy [19]. In Buenos Aires, a high overall mortality of 31.0% was reported among 100 children over a 3-year period. The deaths were associated with admission to ICU, primary bacteraemia or multidrug-resistant isolates [11]. However, in our study those who were admitted to the ICU prior to PABSI diagnosis had a lower mortality at 14 days, which may be the result of early initiation of effective empiric antibiotics, most likely meropenem which was the antibiotic commonly used as an empiric antibiotic in severely ill patients admitted to the ICU. The lower mortality in our study is likely to have been the result of the majority, (50/91(54.9%), of BSI events receiving appropriate empiric antibiotic therapy, as confirmed in the analysis of the determinants of 14-day mortality. In other studies, MDR PA and carbapenem resistance were found to be associated with mortality [20], but this was not demonstrated in our study.

At RCWMCH, the combination of piperacillin-tazobactam and amikacin is frequently used empirically when HAI is suspected. However, a carbapenem antibiotic, usually meropenem is frequently used with or without vancomycin as empiric antibiotic therapy in children with severe HAI including when septic shock is present. Of the HAI and IPOA isolates, 83.3 and 90.3% respectively, were susceptible to amikacin, whereas 58.3 and 61.3% of the HAI and IPOA isolates respectively, were susceptible to piperacillin-tazobactam. Thus, most PA isolates were susceptible to at least one antibiotic in this empiric combination.

Of 19 isolates that were resistant to meropenem, 10 remained susceptible to ceftazidime, an effective antipseudomonal antibiotic. This implies that ceftazidime can be prescribed in a subset of carbapenem-resistant PA infections, as a colistin-sparing intervention. Furthermore, the polymyxins have a narrow therapeutic window, and the major adverse effects of neurotoxicity and nephrotoxicity, makes the use of alternative agents such as ceftazidime desirable when applicable [21].

### Study limitations

Due to the retrospective study design, there were limitations in the availability and completeness of clinical and laboratory data. Additionally, information on previous antimicrobial exposure was limited to what was documented in the patient hospital records. Our sample size was small and was probably underpowered to explore risk factors associated with 14-day mortality comprehensively. Furthermore, our study did not include an appropriate control group, hence risk factors for PABSI were not evaluated. Person- time was not calculated in this study, hence we were unable to determine whether there was the variation in HAI incidence rate using incidence density estimates.

After the study period, it became known that the only reliable method for antimicrobial susceptibility testing (AST) against colistin is broth microdilution minimum inhibitory concentration (MIC) testing; Vitek®2 may miss colistin resistance. The laboratory began referring clinically significant isolates for MIC testing using broth microdilution in December 2017 (after the study period). Thus, the colistin results available for the study cannot be used to interpret AST results or determine appropriate antibiotic use. The breakpoint changed over time for some of the antibiotics used for treatment in the study. Since the authors did not have access to the actual MIC data, and since this is a retrospective study, we were not able to re-classify the antibiotic interpretation of isolates prior to 2012 using the newer breakpoints. Thus, the study may have under-called resistance to piptazobactam and meropenem/imipenem for the isolates preceding 2012. Since this is a retrospective study, the MIC breakpoints for the control strains were not available from the laboratory.

## Conclusion

The study provides useful insights about PABSI at our institution. The most common presenting symptom was respiratory distress, whilst independent determinants of 14-day mortality were empiric antibiotic therapy to which PA isolates were not susceptible, and infections present on admission to hospital. Further research is required to determine whether the presentation of PABSI, PABSI-associated mortality and the determinants of PABSI mortality differ in other parts of sub-Saharan Africa.

## Supplementary information

**Additional file 1.**

## Data Availability

The datasets used and/or analysed during the current study are available from the corresponding author on reasonable request.

## Abbreviations

BSI: Bloodstream infection
CDW: Central Data Warehouse
CLSI: Clinical and Laboratory Standards Institute
GSH: Groote Schuur Hospital
HAI: Healthcare- associated infection
IPOA: Infection present on admission
MDR: multidrug- resistant
PA: *Pseudomonas aeruginosa*
PABSI: *Pseudomonas aeruginosa* bloodstream infection
PDR: Pan drug-resistant
RCWMCH: Red Cross War Memorial Children’s Hospital
XDR: Extensively drug-resistant
