# Supplementary material for: Pseudomonas aeruginosa bloodstream infection at a tertiary referral hospital for children
Source: BMC Infect Dis. 2020 Oct 7;20:729. doi: 10.1186/s12879-020-05437-1 (PMC7541237; doi:10.1186/s12879-020-05437-1)
Supplement: Supplementary file 1 — Additional file 1. [file 12879_2020_5437_MOESM1_ESM.docx]

**Supplementary file – Study definitions**

BSI was classified as *infection present on admission* (IPOA) if PA was cultured from a blood culture specimen obtained on the day of admission to RCWMCH (calendar day 1), 2 days before admission or the calendar day after admission (calendar day 2) or *healthcare-associated infection* (HAI) if PA was isolated from a blood culture specimen obtained on or after the 3^rd^ calendar day of admission to RCWMCH[1].

*Date of onset of PABSI*: The date on which the first positive blood culture for PA was performed.

*Site of infection:* The clinical site of infection as determined by the attending clinician

*Antipseudomonal antibiotics* included gentamicin and amikacin (aminoglycosides), piperacillin (antipseudomonal penicillin), ciprofloxacin (quinolone), ceftazidime and cefepime (cephalosporins), meropenem and imipenem (carbapenems), and colistin (polymyxin).

A PA isolate was classified as (1) *multidrug-resistant (MDR)* if it was non-susceptible to at least one agent in three or more antipseudomonal antibiotic categories, (2) *extensively drug-resistant (XDR)* if it was non- susceptible to at least one agent in all but two or fewer antipseudomonal antibiotic categories, or (3) *pan drug-resistant (PDR)* if it was non-susceptible to all agents in all antipseudomonal antibiotic categories[2].

*Appropriate empiric antibiotic therapy:* antibiotic therapy with *in vitro* activity against the PA isolate that was commenced at the onset of bloodstream infection before the antibiogram of the isolate was known.

*Definitive antibiotic therapy:* antibiotic therapy with *in vitro* activity against the PA isolate that was administered after the antibiogram of the isolate was known.

*Recurrent bloodstream infection:* The re-isolation of PA on blood culture more than 14 days after completion of effective antibiotic therapy for the initial or previous PABSI.

*Central venous access device (CVAD):* An indwelling venous catheter that was inserted into the central venous system with the catheter tip positioned within the superior/inferior vena cava or right atrium, such as Hickman, Port-A-Cath, or central venous pressure (CVP) catheters

*Fever:* An axillary temperature greater or equal to 38 degrees Celsius.

*Anaemia:* Blood haemoglobin concentration <11 g/dl [3].

the onset of bloodstream infection before the antibiogram of the isolate was known.

HIV status was classified as (1) *HIV-infected* in a child <18 months of age with a positive HIV DNA PCR result confirmed by either a quantitative HIV RNA PCR or repeat HIV DNA PCR on a separate sample, or a child ≥18 months of age with 2 positive serological test results (HIV ELISA or HIV rapid test) or a positive HIV DNA PCR result confirmed by either a quantitative HIV RNA PCR or repeat HIV DNA PCR test, (2) *HIV-uninfected* in a child with a negative HIV serological test (HIV ELISA or HIV rapid test) or a negative virological test for HIV (e.g. HIV DNA PCR) or (3) *Unknown* in a child with no history of HIV testing, no record of HIV testing in the NHLS laboratory database and whose mother’s HIV status was unknown.

*Moderate and severe underweight* were defined as weight-for-age Z-score (WAZ) between -2 and -3 standard deviations (SD) and a WAZ <-3 SD below the median WHO growth reference standards, respectively.

*Coagulopathy:* A prothrombin time of ≥2 seconds, an activated partial thromboplastin time of ≥60 seconds or a fibrinogen level of <2 µmol/L.

*Respiratory failure:* the need for mechanical ventilatory support.

*Renal dysfunction:* a serum creatinine concentration above the normal age-related range [4, 5].

*Liver dysfunction:* a ≥2-fold increase of serum aspartate aminotransferase and/or serum alanine aminotransferase concentration and/or a total bilirubin in a child more than 28 days old of >70 µmol/L [6, 7].

*Shock*: The presence of any one of the following criteria – hypotension for age; or any two of the following signs of inadequate tissue perfusion such as prolonged capillary refill, oliguria, metabolic acidosis or elevated tissue lactate.

References

1. Centres for Disease Control and Prevention. Identifying healthcare-associated infections (HAI) for NHSN surveillance. <https://www.cdc.gov/nhsn/pdfs/pscmanual/2psc_identifyinghais_nhsncurrent.pdf>. 2017. Accessed 25 June 2020.

2. Magiorakos AP, Srinivasan A, Carey RB, Carmeli Y, Falagas ME, Giske CG, et al. Multidrug-resistant, extensively drug-resistant and pandrug-resistant bacteria: an international expert proposal for interim standard definitions for acquired resistance. Clin Microbiol Infect. 2012 Mar;18(3):268-81. PubMed PMID: 21793988.

3. World Health Organization. (‎2015)‎. The global prevalence of anaemia in 2011. World Health Organization. <https://apps.who.int/iris/handle/10665/177094>. Accessed 25 June 2020

4. Boer DP, de Rijke YB, Hop WC, Cransberg K, Dorresteijn EM. Reference values for serum creatinine in children younger than 1 year of age. Pediatric nephrology. 2010;25(10):2107-13.

5. Pottel H, Vrydags N, Mahieu B, Vandewynckele E, Croes K, Martens F. Establishing age/sex related serum creatinine reference intervals from hospital laboratory data based on different statistical methods. Clinica Chimica Acta. 2008;396(1):49-55.

6. Goldstein B, Giroir B, Randolph A. International pediatric sepsis consensus conference: definitions for sepsis and organ dysfunction in pediatrics. Pediatric critical care medicine. 2005;6(1):2-8.

7. Dellinger RP, Levy MM, Rhodes A, Annane D, Gerlach H, Opal SM, et al. Surviving Sepsis Campaign: international guidelines for management of severe sepsis and septic shock, 2012. Intensive care medicine. 2013;39(2):165-228.
